# DNA methylation-regulated YTHDF2 correlates with cell migration and immune cell infiltration in glioma

**DOI:** 10.18632/aging.204104

**Published:** 2022-06-02

**Authors:** Xiulin Jiang, Xi Chen, Xiaobin Huang, Chunyan Wang, Chenyang Wang, Chenglong Pan, William C. Cho, Zhi Nie, Jun Pu, Weixiang Wang

**Affiliations:** 1 Kunming College of Life Science, University of Chinese Academy of Sciences, Beijing 100049, China; 2 Department of Neurosurgery, The Second Affiliated Hospital of Kunming Medical University, Kunming, 650223, China; 3 Department of Pathology, The First Affiliated Hospital of Kunming Medical University, Kunming 650032, Yunnan, China; 4 Department of Clinical Oncology, Queen Elizabeth Hospital, Hong Kong SAR, China; 5 Department of Neurology, The First Affiliated Hospital of Kunming Medical University, Kunming 650032, Yunnan, China; 6 Department of Clinical Laboratory, The People’s Hospital of Lishui, Lishui, Zhejiang 323000, China

**Keywords:** cell proliferation, YTHDF2, DNA methylation, cell migration, immune infiltration

## Abstract

Background: Glioma is a lethal malignant brain tumor, it comprises about 80% of all malignant brain tumours. Mounting evidence has reported that YTHDF2 plays a significant role in the cancer progression. However, the effects of YTHDF2 on the prognosis of low-grade gliomas (LGGs) and its correlation with tumor immune infiltration are unclear. The present study was designed to determine the biological functions of YTHDF2 in glioma and to evaluate the association of YTHDF2 expression with glioma progression.

Methods: Clinical data on patients with glioma were obtained from The Cancer Genome Atlas (TCGA), the Chinese Glioma Genome Atlas (CGGA), the Gene Expression Omnibus (GEO), as well as the Rembrandt and Gravendeel databases. The correlations among YTHDF2 expression, pathological characteristics, glioma progression and clinical outcome were evaluated. In addition, the correlation of YTHDF2 expression with immune cell infiltration was analyzed too.

Results: We found that YTHDF2 was significantly up-regulated in LGGs which correlated with tumor grade and poor prognosis. Interestingly, we showed that YTHDF2 expression in LGG was associated with copy number variation, DNA hypomethylation, and induced transcription factor YY1. Besides, KEGG pathway analysis shows that YTHDF2 mainly participates in the immune response and oncogenic signaling pathway. Additionally, YTHDF2 is positively associated with diverse immune cells infiltration, immune cells, and multiple immune checkpoint molecules. Finally, we confirmed that YTHDF2 was highly expressed in LGGs tissues and correlated with the tumor grade with immunohistochemistry assay. More importantly, our results demonstrated that YTHDF2 was elevated in GBM cells. Knockdown of YTHDF2 significantly inhibits the proliferation and migration of GBM cells.

Conclusion: YTHDF2 correlates with glioma progression and immune cell infiltration, suggesting that YTHDF2 may be a useful prognostic biomarker for glioma.

## INTRODUCTION

Glioma is the major type of the brain malignancy, its incidence rate and mortality continue to rise [1]. Gliomas are mainly comprised of low-grade gliomas (LGGs) and glioblastoma multiform (GBM). Although currently there are different treatments for glioma, the survival rate and prognosis of cancer patients is disappointing [2]. Therefore, identifying new biomarkers is crucial for the management of glioma.

RNA m6A modification is a regular mRNA modification, it was first identified in the 1970s [3]. Accumulating evidence demonstrated that YTHDF2, as a member of m6A reader protein, plays a crucial role in RNA metabolism and cancers progression [4]. For example, it has been confirmed that YTHDF2 was up-regulated in HCC and correlated with adverse clinical outcomes in patients [5]. However, there was no study on its expression level and immune roles in glioma.

In this study, we investigated the YTHDF2 expression among several dataset, including TCGA, CGGA and GEO. Moreover, we would study the YTHDF2 expression regulation. Finally, qRT-PCR and IHC assay were used to validate YTHDF2 expression in glioma cells lines and tissues. Loss of function used to determine the biological function of YTHDF2 in glioma progression.

## MATERIALS AND METHODS

### Analysis of the expression and prognosis of YTHDF2

We employed the TCGA (https://www.cancer.gov/), GEO (https://www.ncbi.nlm.nih.gov/geo/query/acc.cgi), CGGA (http://www.cgga.org.cn/) [6], Rembrandt (http://gliovis.bioinfo.cnio.es/) [7] and Gravendeel (http://gliovis.bioinfo.cnio.es/) [8] to examine the expression pattern, clinical significance and prognosis of YTHDF2 in LGG.

### GSEA analysis and immune cell infiltration analysis

In this study, we used GSEA software to explore the signaling pathway involved by YTHDF2 in glioma. TIMER (https://cistrome.shinyapps.io/timer/) [9]. TIMER was used to determine the relationship between YTHDF2 expression and immune cell infiltration in glioma.

### DNA methylation analysis

MEXPRESS (https://mexpress.be/) is an online tool for DNA methylation database, In our study, we use the SMART (http://www.bioinfo-zs.com/smartapp/), EXPRESS (https://mexpress.be/), and MethSurv (https://biit.cs.ut.ee/methsurv/) databases to examine the relationship between the YTHDF2 expression and CNV, DNA methylation in LGG [10–12].

### Drug sensitivity analysis

In this study, we used the GDSC and CTRP databases to explore the correlations between YTHDF2 and various drug sensitivity [13, 14].

### Cell culture and real-time PCR

The glioma cell line was purchased from the cell bank of Kunming Institute of Zoology and cultured in DMEM medium (Corning, USA) supplemented with 10% fetal bovine serum (FBS) and 1% penicillin/streptomycin. The RT-PCR primers shown in the following, YTHDF2-F: AGCCCCACTTCCTACCAGATG, YTHDF2-R: TGAGAACTGTTATTTCCCCATGC; β-actin-F: CTTCGCGGGCGACGAT, β-actin-R: CCATAGGAATCCTTCTGACC. The expression quantification was obtained with the 2^−ΔΔCt^ method. The shRNA primer used in this study following: shYTHDF2-1: CCTACTTACCCAGTTACTACA, shYTHDF2-2: GCTCTGGATATAGTAGCAATT.

### Cell proliferation, cell migration and IHC assay

Cell proliferation, cell migration and IHC assay was performed as previously described [15]. Cell proliferation and cell migration assay used to determine the function of YTHDF2 on glioma cell migration and proliferation. IHC assay used to explore the expression level of YTHDF2 in glioma tissues.

## RESULTS

### Expression and prognostic values of YTHDF2 in human cancers

**Figure 1:**
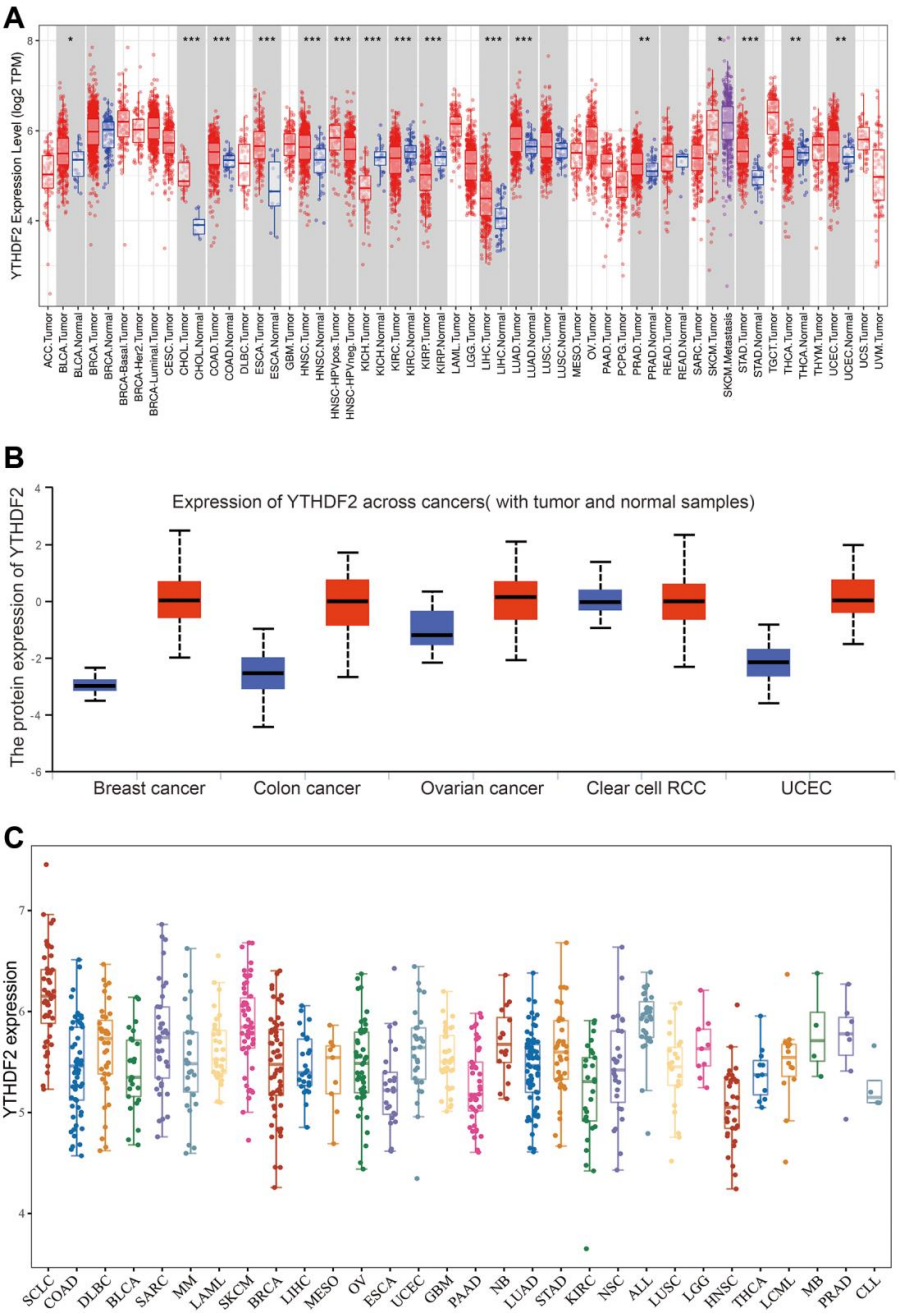
**The expression of YTHDF2 in human cancer.** (**A**) The expression of YTHDF2 in pan-cancer examine by TIMER database. (**B**) The protein level of YTHDF2 in diverse cancer examine by the UALCAN database. (**C**) The expression of YTHDF2 in diverse cancer cell lines examined by CCLE database.

To examine the expression pattern of YTHDF2 in pan-cancer, we used the TIMER database analysis it’s expression pattern, the result demonstrated that YTHDF2 was significantly over-expression in BLCA, CHOL, COAD, ESCA, HNSC, KICH, KIRP, KIRC, LIHC, LUAD, PRAD, SKCM, STAD, THCA and UCEC (Figure 1A). Similar to YTHDF2 mRNA, the protein level of YTHDF2 was highly in BRCA, COAD, OV, KIRC, and UCEC (Figure 1B). Moreover, we employ the CCLE databases to examine YTHDF2 expression in various cancer cells lines. Results suggested that YTHDF2 was elevated in different cancer cell lines (Figure 1C).

**Figure 2:**
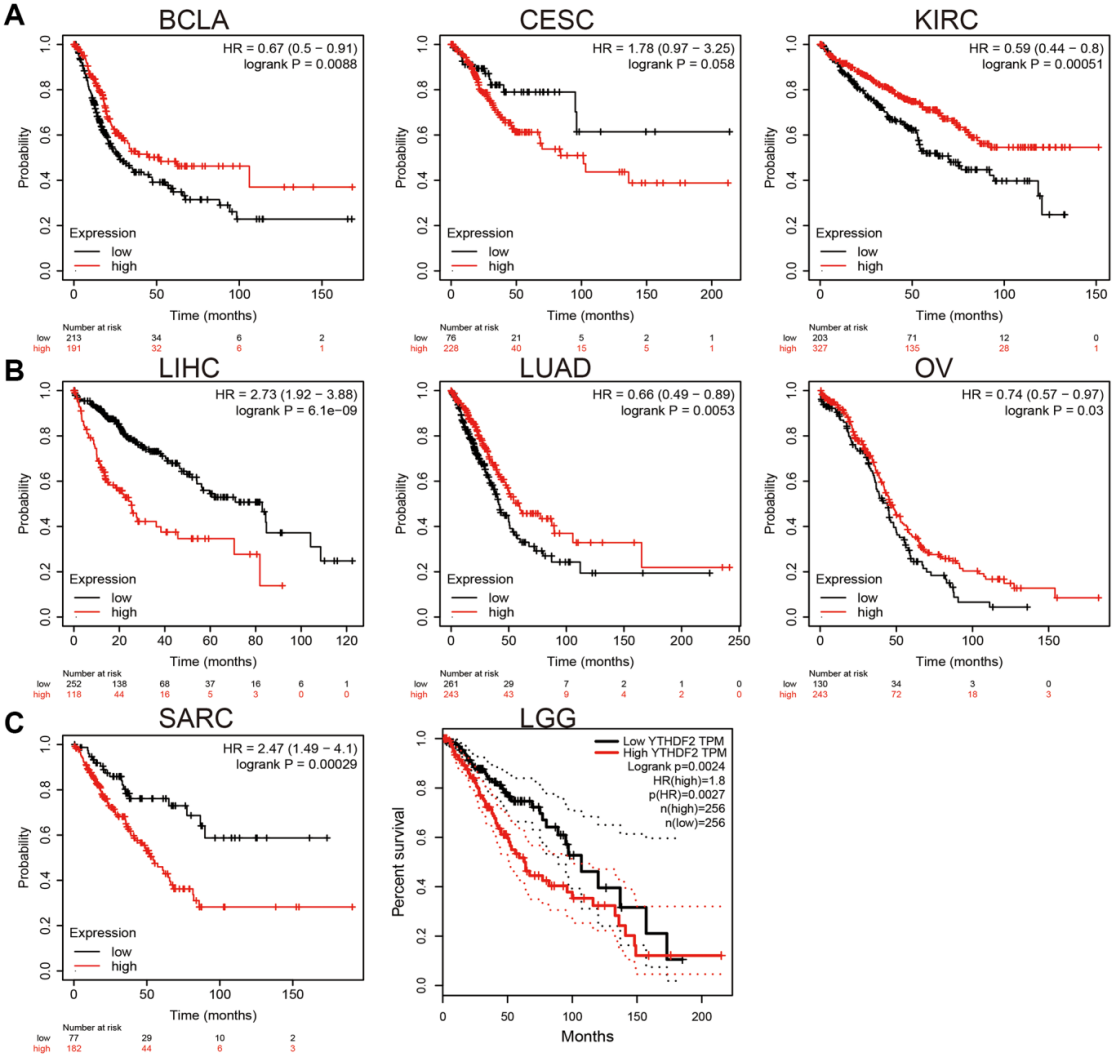
**The prognosis value of YTHDF2 in human cancer.** (**A**) The prognosis value of YTHDF2 in BLCA, CESC, and KIRC was examined by the km plot database. (**B**) The prognosis value of YTHDF2 in LIHC, LUAD, and OV was examined by the km plot database. (**C**) The prognosis value of YTHDF2 in SARC and LGG was examined by the km plot database.

We employed KM plot to explore the prognostic value of YTHDF2 expression in human cancer. Our results confirmed that higher level of YTHDF2 overexpression closely correlates to the poor clinical prognosis of LIHC, SARC, and LGG. On the contrary, up-regulation of YTHDF2 correlated with a better prognosis in BLCA, CESC, KIRC, LUAD, and OV (Figure 2).

### YTHDF2 was up-regulated in LGG

**Figure 3:**
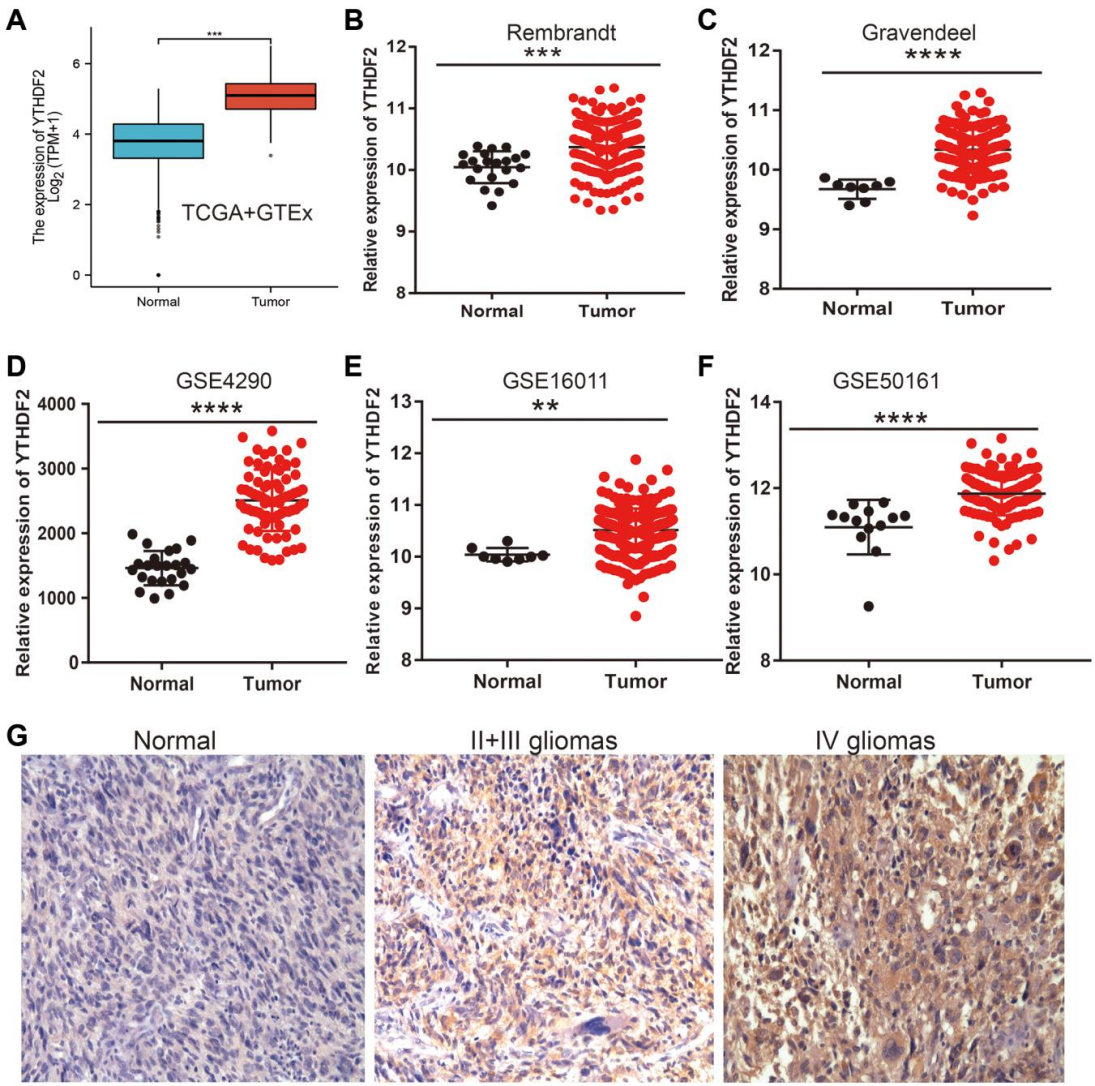
**YTHDF2 was the high expression in LGG.** (**A**–**F**) The YTHDF2 expression is significantly regulated in glioma examined by the TCGA (**A**), Rembrandt (**B**), Gravendeel (**C**), and GEO datasets (**D**–**F**). (**G**) The immunohistochemistry detection of YTHDF2 in Normal brain tissue, LGG, and HGG.

To determine the expression of YTHDF2 in LGG, we adopt the public database to examine the expression of YTHDF2 in glioma tissues and normal group. Results found out that YTHDF2 was overexpressed in the LGG tissues (Figure 3A–3C). Similar results were also obtained from the diverse GEO datasets (Figure 3D–3F). Furthermore, the results demonstrated that the YTHDF2 protein level was increased in glioma tissues (Figure 3G). Collectively, these data indicate that YTHDF2 was up-regulated in glioma tissues and high expression of YTHDF2 was significantly correlated with the tumor grade of LGG.

### Relationship between YTHDF2 expression and clinical features

**Figure 4:**
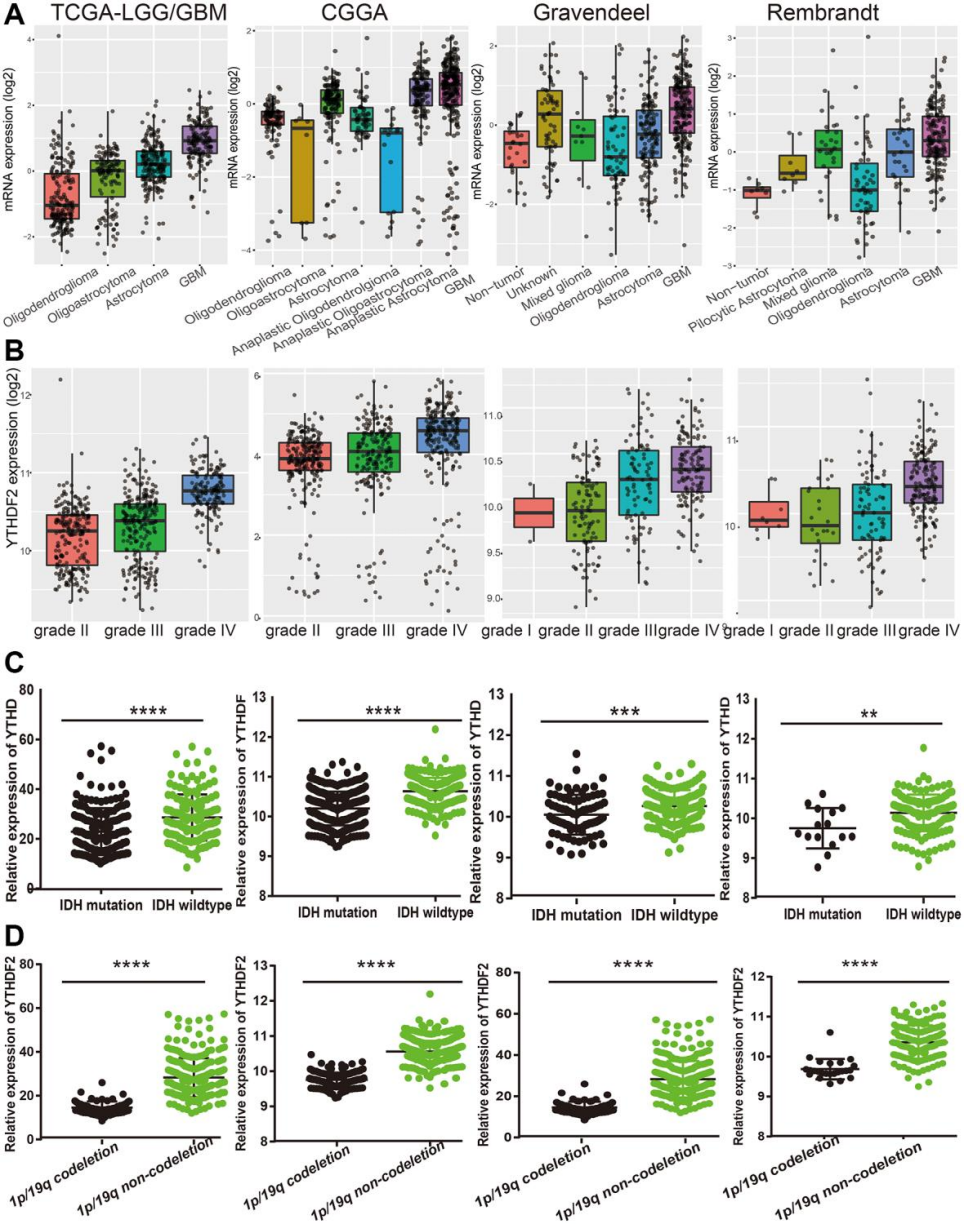
**The correlation between the YTHDF2 expression and clinical information in LGG.** (**A**) The expression of YTHDF2 in diverse histology of glioma based on TCGA, CGGA, Rembrandt, and Gravendeel databases. (**B**) The expression of YTHDF2 in diverse tumor grades of glioma based on TCGA, CGGA, Rembrandt, and Gravendeel databases. (**C**) The expression of YTHDF2 in diverse IDH mutations of glioma based on TCGA, CGGA, Rembrandt, and Gravendeel databases. (**D**) The expression of YTHDF2 in diverse 1p/19q codeletion of glioma based on TCGA, CGGA, Rembrandt, and Gravendeel databases.

Next, we examine the relationship between YTHDF2 expression and clinical features in LGG. Results confirmed that YTHDF2 differential expression in different histology subtypes (Figure 4A). Additionally, the YTHDF2 expression level was increased with the tumor grade elevated (Figure 4B). We also found that YTHDF2 was markedly reduced in the IDH mutation group and the 1p/19q chromosome co-deletion group (Figure 4C, 4D). Interestingly, YTHDF2 was down-regulation in patients over 40 years old (Supplementary Figure 1A, 1B).

### Prognostic value of YTHDF2

**Figure 5:**
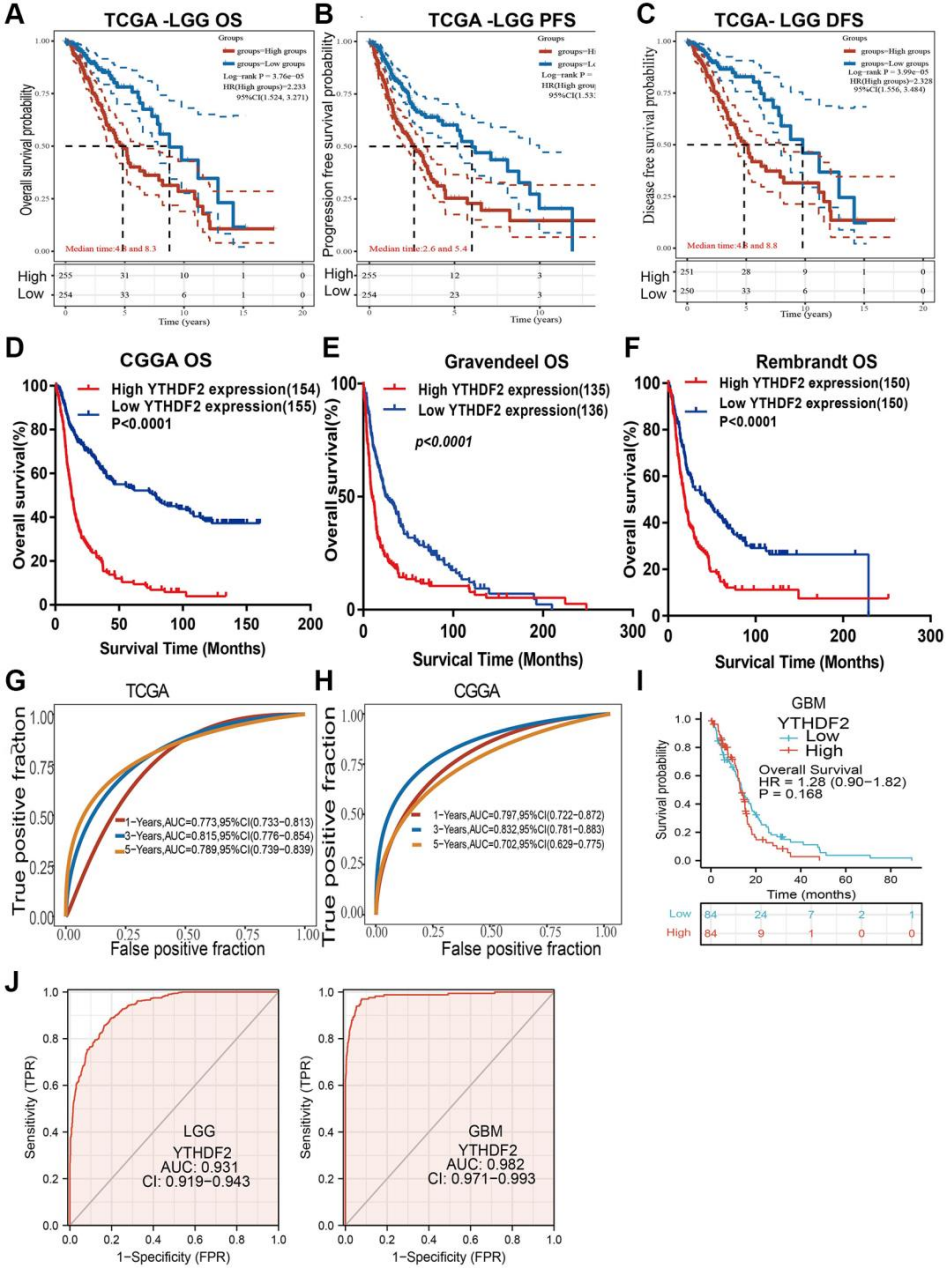
**The prognosis of YTHDF2 in LGG.** (**A**–**F**) The prognosis of YTHDF2 in LGG was examined by TCGA, CGGA, Rembrandt, and Gravendeel databases. (**G**, **H**) ROC analyses revealed the predictive value of YTHDF2 in glioma based on TCGA-LGG, CGGA, and TCGA-GBM. (**I**) The prognostic value of YTHDF2 in GBM. (**J**) The diagnostic value of YTHDF2 in GBM and LGG.

We found that up-regulation of YTHDF2 was correlated with the unfavorable prognosis of glioma patients in different datasets (Figure 5A–5F). Additionally, we perform the ROC curves analysis showed that the area under the curve (AUC) of YTHDF2 were 0.773, 0.815, and 0.789 for the TCGA-LGG datasets (Figure 5G), 0.797, 0.832, and 0.702 for the CGGA datasets (Figure 5H) in 1, 3, and 5 years, respectively. In GBM, the overall of patients no significant difference between high and low YTHDF2 group (Figure 5I). ROC curve of YTHDF2 show an AUC value of 0.982 and 0.931 based on the TCGA-GBM and TCGA-LGG dataset (Figure 5J).

### DNA methylation analysis

**Figure 6:**
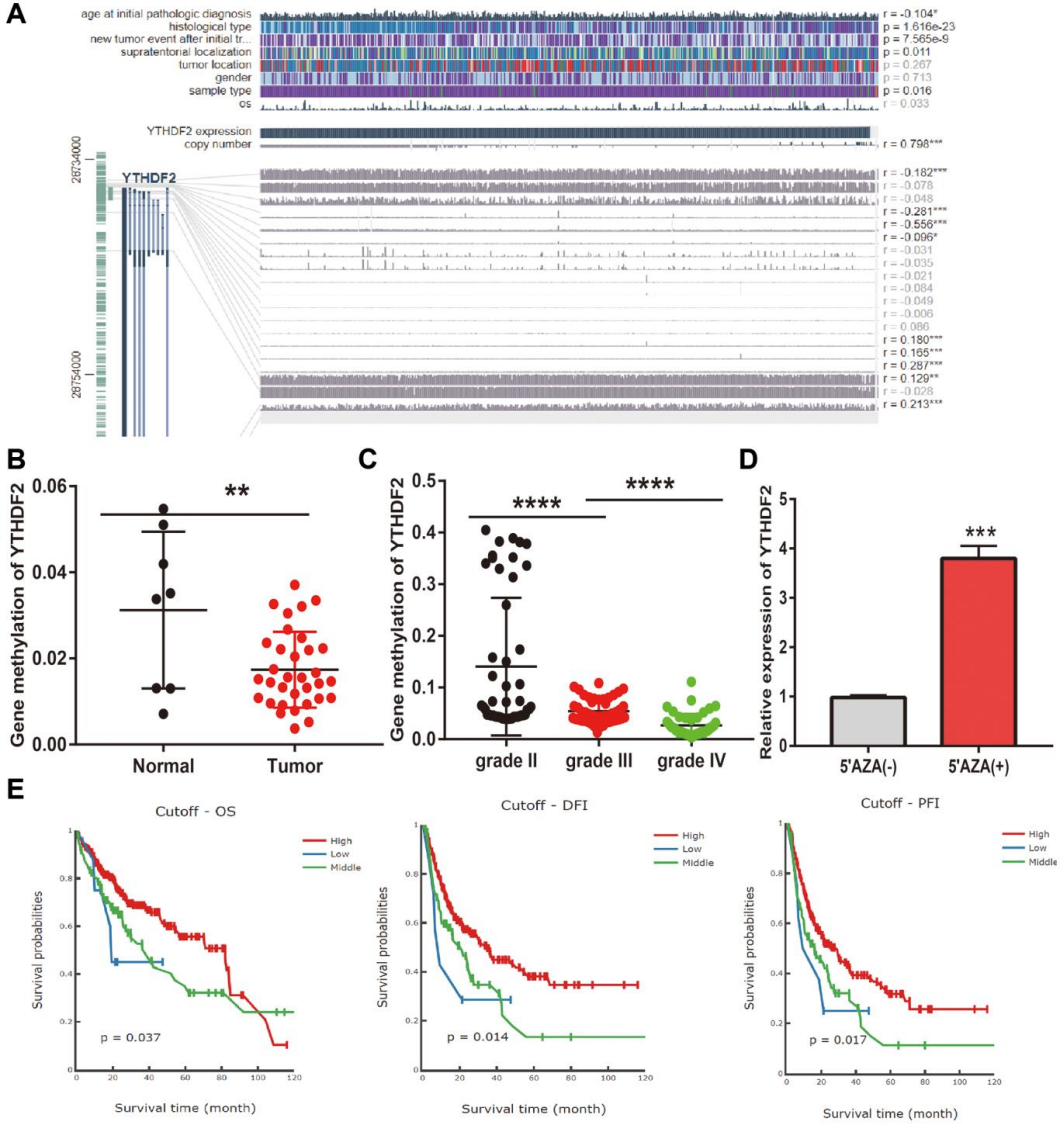
**Analysis of the YTHDF2 methylation level.** (**A**) The correlation between CNV, DNA methylation, and YTHDF2 expression is examined by EXPRESS. (**B**) The DNA methylation level of YTHDF2 in LGG. (**C**) The DNA methylation level of YTHDF2 in diverse tumor grades of LGG based on CGGA dataset. (**D**) The expression of YTHDF2 in U251 cells after using 5-AZA treatment was examined by qRT-PCR assay. (**E**) The prognosis for the methylation level of YTHDF2 in the TCGA-LGG dataset.

DNA methylation plays a crucial role in the regulation of gene expression. To elucidate the abnormal up-regulated mechanisms of YTHDF2 in LGG tissues, we further examine the relationship between DNA methylation, CNV, and the expression of YTHDF2 in gliomas. Firstly, we found that CNV of YTHDF2 was positively correlated with the expression (r = 0.798, *p* < 0.0001) (Figure 6A). Furthermore, we confirmed that various methylation sites in the promoter region of YTHDF2, among these methylation sites, the cg28734000 was negative correlated with the expression of YTHDF2 in LGG (r = 0.–0.556, *p* < 0.0001). CGGA dataset also obtained same results (Figure 6B, 6C). 5-Azacytidine, as an inhibitor of DNA methyltransferase [16], was used to treat the U251 cells, the results show that inhibiting the DNA methylation could significantly enhance YTHDF2 RNA levels in U251 cells (Figure 6D). Finally, we uncovered that lower methylation levels on the cg28734000 site correlate with adverse clinical outcomes in the TCGA-LGG dataset examined by the measure database (Figure 6E).

### YY1 activates YTHDF2 expression in glioma cells

**Figure 7:**
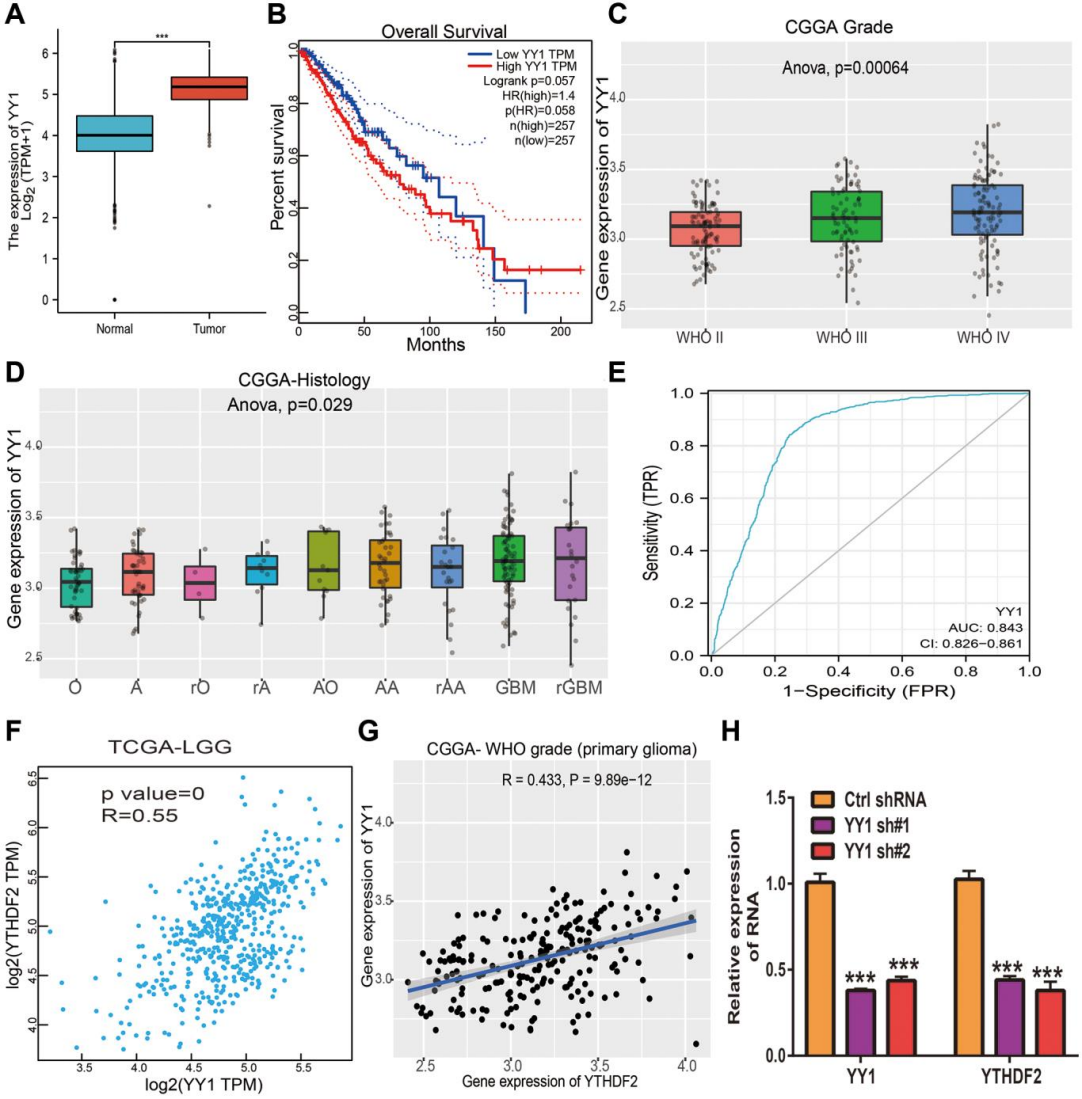
**YY1 activates YTHDF2 expression in glioma cells.** (**A**) The expression of YY1in LGG tissues and normal tissues. (**B**) The prognosis of YY1 in TCGA-LGG was examined by GEPIA. (**C**) The expression of YY1 in diverse tumor grades of LGG examined by CGGA databases. (**D**) The expression of YY1 in diverse histology of in CGGA-LGG databases. (**E**) The ROC curve value of YY1 in TCGA-LGG. (**F**) YY1 expression was strongly positively associated with YTHDF1 expression in TCGA-LGG examined by GEPIA. (**G**) YY1 expression was strongly positively associated with YTHDF1 expression in LGG examined by CGGA. (**H**) The expression of YTHDF2 in U251 cells upon depletion of YY1 was examined by qRT-PCR assay.

In order to determine the aberrant up-regulation of YTHDF2 in LGG, we employed JASPAR, Knock TF, and PROMO algorithms” to predict the potential transcription factors that may bind to YTHDF2 promoters. The results found that there are 4 common transcription factors in both prediction results, including the STAT3, JUN, MAF, and YY1. Then, we used the GEPIA database to analyze the correlation between these transcription factors and YTHDF2 expression in glioma samples based on the TCGA and CGGA dataset and found that only YY1 was positively associated with YTHDF2 expression. We also found that among these transcription factors, only YY1 was up-regulated in LGG specimens (Figure 7A). Elevated YY1 expression related to poor OS in TCGA-LGG (Figure 7B). Additionally, we found that YY1 expression was significantly increased in diverse WHO grade (*p* = 0.0006), (Figure 7C), and histology (*p* = 0.02, Figure 7D). ROC curve analysis of YY1 showed an AUC value of 0.843 in LGG patients (Figure 7E). By analyzing the TCGA LGG and CGGA datasets, we found that YY1 was strongly correlated with YTHDF2 expression (Figure 7F, 7G). Finally, to validate the above results, we performed the qRT-PCR assay, we found out that knock-down of YY1 was markedly reduced the YTHDF2 levels in U251 cells (Figure 7H). These results confirmed that YY1 can increase YTHDF2 expression via modulating the transcription process of YTHDF2 in glioma cells.

### GSEA analysis

**Figure 8:**
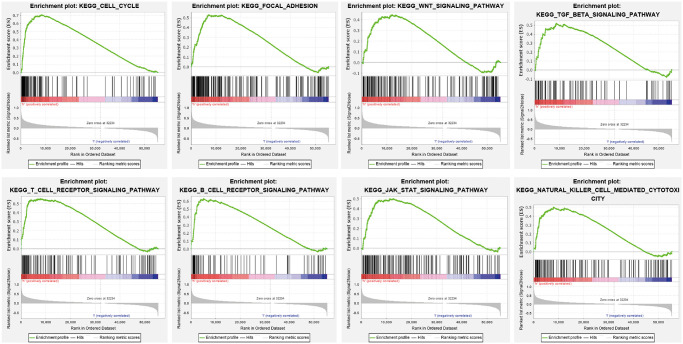
**KEGG signaling pathway explored by GSEA software.** The Cell Cycle, Focal adhesion, Wnt signal pathway JAK- STAT3- signal pathway, T cell receptor signal pathway, and B cell receptor signal pathway in LGG were examined by GSEA software.

To determine the signaling pathway affected by YTHDF2 in LGG, we performed the GSEA enriched analysis using the GSEA software, results showed that YTHDF2 was mainly involved in the cell cycle, Focal adhesion, Wnt signal pathway, JAK- STAT3-signal pathway (Figure 8).

### Association between YTHDF2 expression and immune cell infiltration

**Figure 9:**
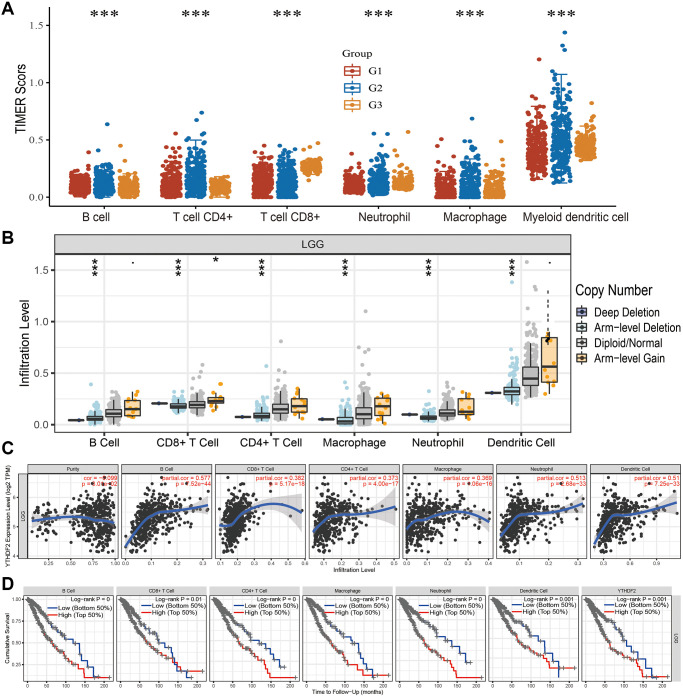
**Analysis of the correlation between YTHDF2 expression and diverse immune cell infiltration**. (**A**) The level of diverse immune infiltration in different tumor grades of LGG. (**B**) The correlation between YTHDF2 expression and somatic copy number alterations. (**C**) The correlation between YTHDF2 expression and diverse immune cell infiltration. (**D**) The B cells, CD4+ T cells, CD8+ T cells, dendritic cells, Macrophages, and Neutrophils are correlated with the cumulative survival rate in LGG.

Firstly, we analysis the immune infiltration levels based on the different tumor grade, the result demonstrated that different immune infiltration levels observed in the different grade tumor samples (Figure 9A). Furthermore, we found that CNV of YTHDF2 was markedly affect the immune infiltration levels of different immune (Figure 9B). Finally, we found that YTHDF2 was markedly correlated with B cells (cor = 0.577, *P* = 7.52e-44), CD8+ T cell (cor = 0.382, *P* = 5.17e-18), CD4+ T cells (cor = 0.373, *P* = 4.00e-17), macrophages (cor = 0.369 *P* = 1.06e-16), neutrophils (cor = 0.513, *P* = 2.68e-33), and dendritic cells (cor = 0.51, *P* = 7.25e-33) (Figure 9C). We also uncovered that YTHDF2 was positive correlated with the abundances of Th2 cells, T helper cells Tgd, Eosinophils Macrophages, and Neutrophils, negatively associated with the abundances of DCNK, CD56bright cells, and pDC in LGG (Supplementary Figure 1C). In GBM dataset, YTHDF2 was positive correlated with the abundances of Th2 cells, T helper cells, Tgd, and NK cells, negatively associated with the abundances of T cells, DC Macrophages, DC, Neutrophils, and Cytotoxic cells (Supplementary Figure 1D). The Cox model confirmed that B cells and YTHDF2 were significantly related to the poor OS in LGG patients (Figure 9D).

**Figure 10:**
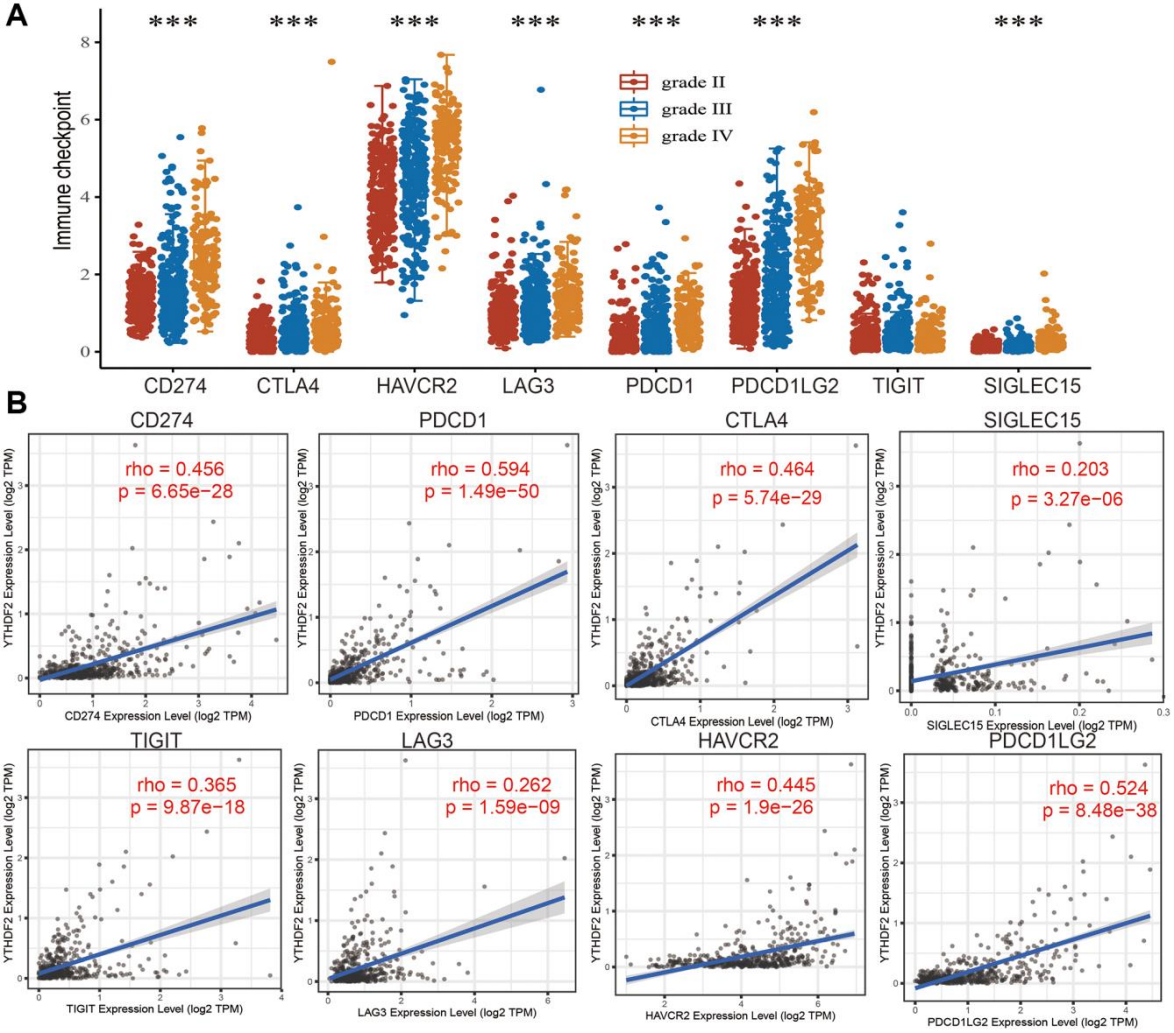
**The association between YTHDF2 expression and immune checkpoints.** (**A**) The expression of immune checkpoints-related genes is different from the WHO grade of LGG. (**B**) The correlations between YTHDF2 expression and diverse immune checkpoints related genes were examined by the TIMER database.

We analyzed the immune checkpoints related gene expression distribution in WHO grade II, III and IV gliomas tissues, the result demonstrated that immune checkpoints related gene differential expression in diverse samples (Figure 10A). In addition, we also analyzed the relationship between expression of YTHDF2 and immune checkpoint-related genes by Pearson correlation analysis in LGGs. Result shown that YTHDF2 expression was significantly positively correlated with CD274 (cor = 0.456, *P* = 6.65e-28), CTLA4 (cor = 0.464, *P* = 5.74e-29), HAVCR2 (cor = 0.445, *P* = 1.9e-26), LAG3 (cor = 0.262, *P* = 1.59e-09), PDCD1 (cor = 0.594, *P* = 1.49e-50), PDCD1LG12 (cor = 0.524, *P* = 8.48e-38), TIGIT (cor = 0.365, *P* = 9.87e-18), SIGLEC15 (cor = 0.203, *P* = 3.27e-06) (Figure 10B).

### YTHDF2 expressions were correlated with diverse immune cell type markers

We explored the relationship between YTHDF2 expression and various immune cells as well as immune markers in LGG by employing the TISIDB and TIMER database, the TISIDB analysis result indicated that YTHDF2 expression was significantly correlated with 28 tumor-infiltrating lymphocytes (Table 1). Furthermore, the results indicated that YTHDF2 expression was positively correlated with the immune cell marker, in LGG (Table 2).

**Table 1:** Analysis the correlation between YTHDF2 expression and diverse lymphocyte infiltration in LGG examined by TISIDB database.

	**LGG**	
	***r***	***p***
Activated CD8 T cell (Act CD8)	0.43	^***^
Central memory CD8 T cell (Tcm CD8)	0.57	^***^
Effector memory CD8 T cell (Tem CD8)	0.693	^***^
Activated CD4 T cell (Act CD4)	0.236	^***^
Central memory CD4T cell (Tcm CD4)	0.614	^***^
Effector memory CD4 T cell (Tem CD4)	0.414	^***^
T follicular helper cell (Tfh)	0.838	^***^
Gamma delta T cell (Tgd)	0.535	^***^
Type 1 T helper cell (Th1)	0.651	^***^
Type 17 T helper cell (Th17)	0.393	^***^
Type 2 T helper cell (Th2)	0.252	^***^
Regulatory T cell (Treg)	0.706	^***^
Activated B cell (Act B)	0.7	^***^
Immature B cell (Imm B)	0.802	^***^
Memory B cell (Mem B)	0.633	^***^
natural killer cell (NK)	0.757	^***^
CD56bright natural killer cell (CD56bright)	0.272	^***^
CD56dim natural killer cell (CD56dim)	0.193	^***^
Myeloid derived suppressor cell (MDSC)	0.869	^***^
Natural killer T cell (NKT)	0.715	^***^
Activated dendritic cell (Act DC)	0.773	^***^
Plasmacytoid dendritic cell (pDC)	0.485	^***^
Immature dendritic cell (iDC)	0.642	^***^
Macrophage (Macrophage)	0.856	^***^
Eosinophil (Eosinophil)	0.453	^***^
Mast (Mast)	0.808	^***^
Monocyte (Monocyte)	0.44	^***^
Neutrophil (Neutrophil)	0.422	^***^

**Table 2:** Analysis the correlation between YTHDF2 expression and diverse immune cells markers in LGG examined by TIMER database.

**Description**	**Gene markers**	**None**		**Purity**	
		**Cor**	***P***	**Cor**	***P***
CD8+ T cell	CD8A	0.649	^***^	0.183	^***^
	CD8B	0.491	^***^	0.137	^***^
T cell	CD3D	0.794	^***^	0.394	^***^
	CD3E	0.837	^***^	0.459	^***^
	CD2	0.839	^***^	0.462	^***^
B cell	CD19	0.386	^***^	0.352	^***^
	CD79A	0.284	^***^	0.434	^***^
	CD27	0.356	^***^	0.182	^**^
Monocyte	CD14	0.42	^***^	0.29	^***^
TAM	CCL2	0.46	^***^	0.229	^***^
	CD68	0.468	^***^	0.287	^***^
	IL10	0.444	^***^	0.238	^***^
M1 Macrophage	CD80	0.48	^***^	0.223	^***^
	IRF5	0.399	^***^	0.237	^***^
	IL6	0.456	^***^	0.298	^***^
	CD64	0.568	^***^	0.345	^***^
	CD163	0.435	^***^	0.219	^***^
M2 Macrophage	VSIG4	0.285	^***^	0.198	^***^
	MS4A4A	0.424	^***^	0.236	^***^
	CD66b	0.674	^***^	0.427	^***^
	CD11b	0.457	^***^	0.218	^***^
Neutrophil	CD15	0.541	^***^	0.328	^***^
	KIR2DL1	0.153	^***^	0.012	^*^
	KIR2DL3	0.301	^***^	0.128	^***^
Natural killer cell	KIR3DL2	0.178	^***^	0.012	0.37
	CD1C	0.436	^***^	0.237	^***^
	NRP1	0.436	^***^	0.319	^***^
	ITGAX	0.351	^***^	0.198	^***^
Dendritic cell	TBX21	0.578	^***^	0.389	^***^
	STAT4	0.188	^***^	0.023	^***^
	STAT1	0.5531	^***^	0.428	^***^
	GATA3	0.492	^***^	0.398	^***^
	STAT6	0.463	^***^	0.238	^***^
Th1	BCL6	−0.126	^**^	−0.01	0.23
	STAT3	0.422	^***^	0.287	^***^
Th2	IL17A	0.328	^***^	0.195	^***^
	FOXP3	0.471	^***^	0.289	^***^
	CD25	0.65	^***^	0.467	^***^
Tfh	CCR8	0.245	^***^	0.118	^***^
	STAT5B	0.563	^***^	0.398	^***^

### Drug sensitivity analysis

**Figure 11:**
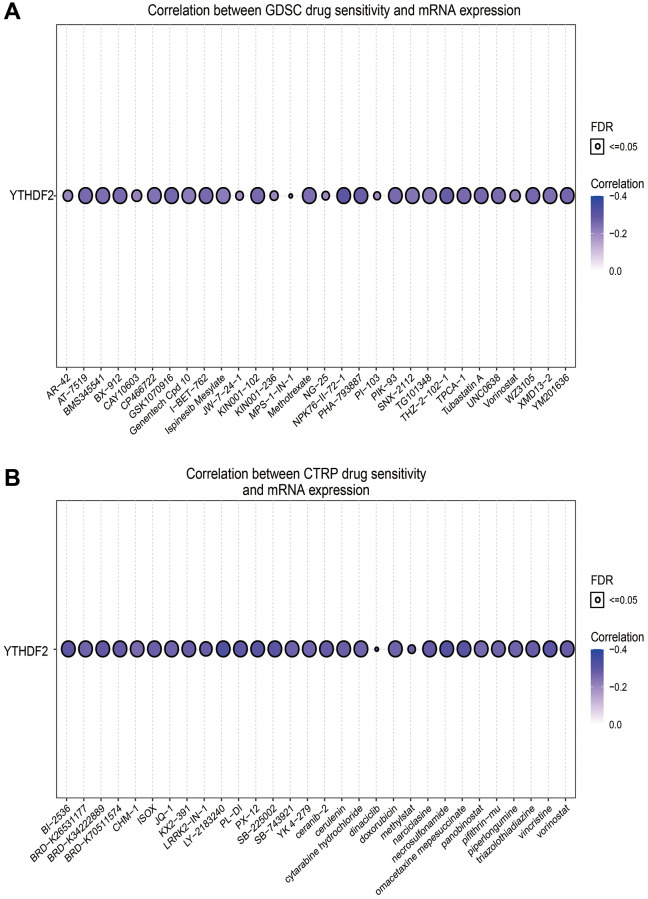
**The correlation between YTHDF2 expression and drug sensitivity in LGG.** (**A**) The correlation between the YTHDF2 expression and drug sensitivity was examined by the GDSC database. (**B**) The correlation between the YTHDF2 expression and drug sensitivity was examined by the CTRP database.

Given that YTHDF2 has a potential driving effect on the progression of LGG, we used the GSCA tools to analyze the relationship between the YTHDF2 expression and drug sensitivity. Results indicated that YTHDF2 expression positive correlated with the drug sensitivity of XAV939, 17-AAG, Cetuximab, TGX221, Erlotinib, and Trametinib (r > 0.16, *p* < 0.0001), negative related to the drug sensitivity of NPK76-II-72-1, THZ-2-102-1, PHA-793887, WZ3105, YM201636, GSK1070916, Tubastatin A, I-BET-762, KIN001-102, AT-7519, PIK-93, TPCA-1, BX-912 and UNC0638 in GDSC and CTRP database (r < −0.25, *p* < 0.0001) (Figure 11A, 11B and Tables 3, 4).

**Table 3:** Analysis the correlation between YTHDF2 expression and diverse drug sensitivity by using GDSC database.

**Symbol**	**Drug**	**Cor**	**Fdr**
YTHDF2	NPK76-II-72-1	−0.30021	1.36E-19
YTHDF2	THZ-2-102-1	−0.27868	2.81E-16
YTHDF2	PHA-793887	−0.2772	1.13E-16
YTHDF2	WZ3105	−0.26037	1.35E-14
YTHDF2	YM201636	−0.25939	4E-14
YTHDF2	GSK1070916	−0.25896	4.85E-14
YTHDF2	Tubastatin A	−0.25819	2.48E-14
YTHDF2	I-BET-762	−0.25474	3.33E-14
YTHDF2	KIN001-102	−0.25473	5.14E-14
YTHDF2	AT-7519	−0.25449	6.2E-14
YTHDF2	PIK-93	−0.25431	4.41E-14
YTHDF2	TPCA-1	−0.25414	4.74E-14
YTHDF2	BX-912	−0.25382	5.31E-14
YTHDF2	UNC0638	−0.25122	3.67E-14
YTHDF2	XAV939	0.188419	2.23E-07
YTHDF2	17-AAG	0.184788	2.74E-07
YTHDF2	Cetuximab	0.172627	3.9E-06
YTHDF2	TGX221	0.172132	0.003902
YTHDF2	Erlotinib	0.167506	0.011063
YTHDF2	Trametinib	0.160217	6.33E-06

**Table 4:** Analysis the correlation between YTHDF2 expression and diverse drug sensitivity by using CTRP database.

**Symbol**	**Drug**	**Cor**	**Fdr**
YTHDF2	GSK-J4	−0.40462	0.004391
YTHDF2	LY-2183240	−0.32709	1.16E-19
YTHDF2	dinaciclib	−0.32552	1.24E-09
YTHDF2	necrosulfonamide	−0.31263	2.45E-12
YTHDF2	BRD-K30748066	−0.31086	0.057866
YTHDF2	omacetaxine mepesuccinate	−0.30419	2.43E-12
YTHDF2	PX-12	−0.30395	1.07E-16
YTHDF2	vincristine	−0.30344	1.03E-17
YTHDF2	SB-225002	−0.3032	2.88E-17
YTHDF2	tivantinib	−0.29689	3.47E-08
YTHDF2	BI-2536	−0.29528	1.56E-16
YTHDF2	PL-DI	−0.29412	1.38E-15
YTHDF2	BRD-K34222889	−0.28871	2.71E-15
YTHDF2	narciclasine	−0.28509	1.41E-14
YTHDF2	BRD-K70511574	−0.28292	1.24E-14
YTHDF2	ceranib-2	−0.28174	6.94E-15
YTHDF2	vorinostat	−0.28052	2.52E-14
YTHDF2	triazolothiadiazine	−0.28049	6.15E-15
YTHDF2	dasatinib	0.18171	7.07E-06
YTHDF2	saracatinib	0.179659	1.13E-05
YTHDF2	VAF-347	0.13649	0.018721
YTHDF2	abiraterone	0.101956	0.445064

### Knock down of YTHDF2 inhibited cell proliferation and migration

**Figure 12:**
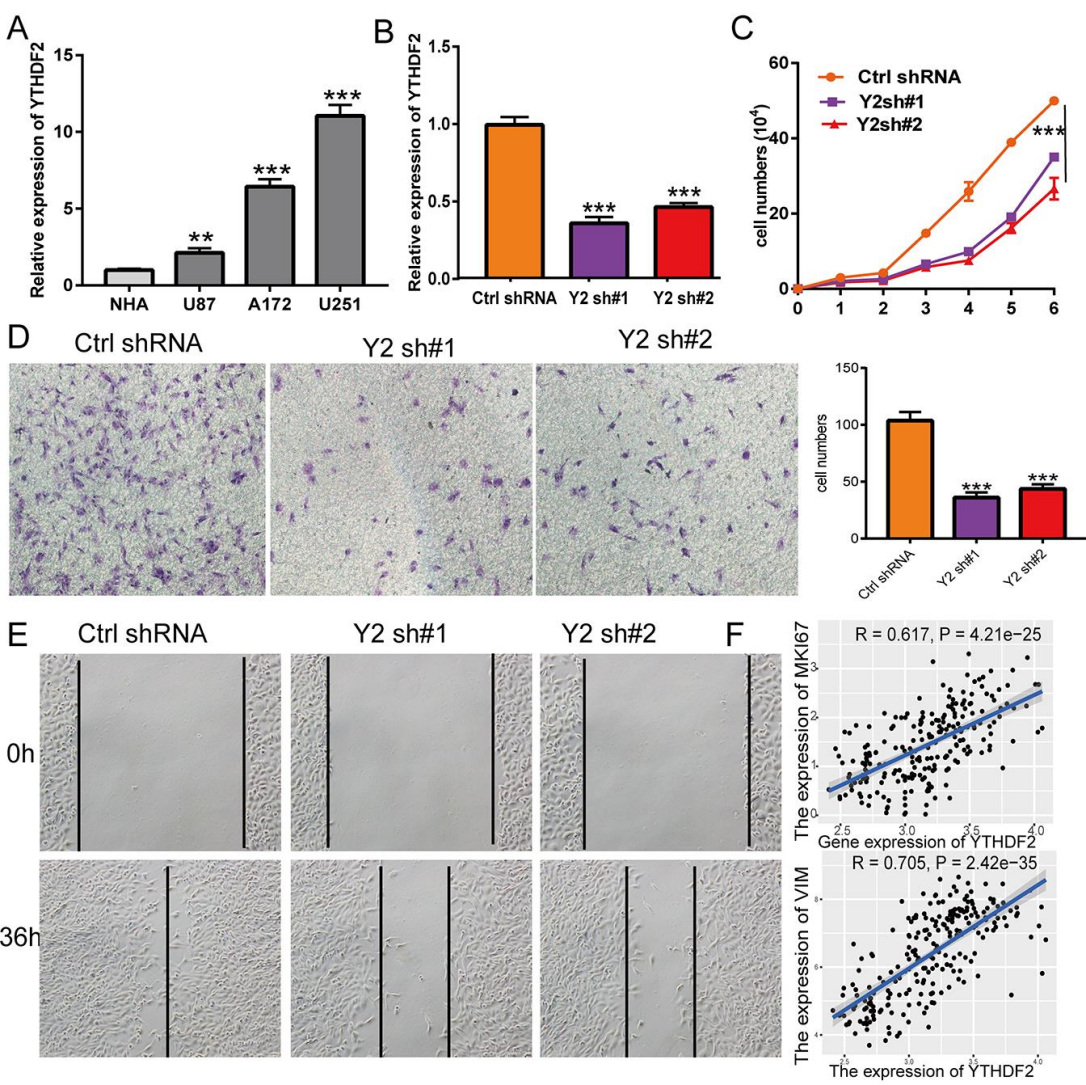
**Depletion of YTHDF2 inhibits GBM cell proliferation and migration.** (**A**) The expression of YTHDF2 in normal human astrocytes cells (NHA) and GBM cell lines (U87, A172, and U251). (**B**) The YTHDF2 knockdown efficiency in U251 was verified by qRT-PCR assay. (**C**) YTHDF2 knockdown significantly inhibited U251 cell proliferation examined by growth curve assay. (**D**) YTHDF2 knockdown significantly inhibited U251 cell migration examined by transwell assay. (**E**) YTHDF2 knockdown significantly inhibited U251 cell migration examined by wound healing assay. (**F**) Pearson correlation analysis the correlation between YTHDF2 and MKI67, VIM expression in TCGA LGG.

To examine the function of YTHDF2 in LGG, we used the qRT-PCR assay to detect the YTHDF2 expression in glioma cell lines. The results show that YTHDF2 was the high expression in glioma cell lines, especially in U251cells (Figure 12A). Owing to the higher expression of YTHDF2 in glioma cell lines, we construction the YTHDF2 knockdown cells and using the qRT-PCR assay verify the knockdown efficiency (Figure 12B). By performing the loss of function for YTHDF2 in U251cell, we found that YTHDF2 knock down was significantly inhibited the cell proliferation of U251 cells by growth curve assay (Figure 12C). Additionally, the decreased migration ability of U251 cells was observed in the knockdown of YTHDF2 (Figure 12D, 12E). We also uncover that YTHDF2 was strongly correlated with Ki-67 expression (r = 0.617, *P* = 4.21e-25) and vimentin (r = 0.705, *P* = 2.42e-35) (Figure 12F).

## DISCUSSION

YTHDF2, a member of the m6A reader proteins containing the YT521-B homology (YTH) domain family, has been reported to play important roles in cancer progression. We found that YTHDF2 expression was up-regulated in glioma and its high expression was correlated with the tumor grade and poor prognosis in LGG. ROC curve confirmed that the YTHDF2 expression level has diagnostic value for gliomas.

Our KEGG pathway analysis shows that YTHDF2 expression was highly correlated with immune response and oncogenic signaling pathway. DNA methylation and transcription factor were reported that play an essential role in regulating gene expression.

Here we found that YTHDF2 expression was strongly negatively correlated with DNA methylation, and we use 5-aza-2’-deoxycytidine can restore YTHDF2 expression. Therefore, DNA hypomethylation may be a cause for YTHDF2 upregulated in LGG. At the transcription level, YY1 could increase the expression of YTHDF2 by modulating the transcription process of YTHDF2.

Previous study confirmed that YTHDF2 was crucial for maintain the stability of RNA. In this study, we confirmed that YTHDF2 was mainly involved in the cell cycle, Focal adhesion, Wnt signal pathway, JAK- STAT3-signal pathway.

Mounting evidence has demonstrated that the TME plays an important role in glioma progression [17]. Here we found that a strong positive relationships between YTHDF2 expression level and various immune cell infiltration. We also investigated the correlations between YTHDF2 expression and immune checkpoint. YTHDF2 levels demonstrated strong correlations with multiple immune checkpoint molecules, such as CD274 and CTLA4.

Finally, we found that YTHDF2 was highly expressed in GBM cells, depletion of YTHDF2 was significantly inhibited the cell proliferation and migration of GBM cells, the IHC assay also demonstrated that YTHDF2 was up-regulation in LGG and correlated with tumor grade.

## Supplementary Materials

Supplementary Figure 1
