# Supplementary material for: DNA methylation-regulated YTHDF2 correlates with cell migration and immune cell infiltration in glioma
Source: Aging (Albany NY). 2022 Jun 2;14(19):7774–93. doi: 10.18632/aging.204104 (PMC9596213; doi:10.18632/aging.204104)
Supplement: Supplementary Figure 1 [file aging-14-204104-s001.pdf]

SUPPLEMENTARY FIGURE

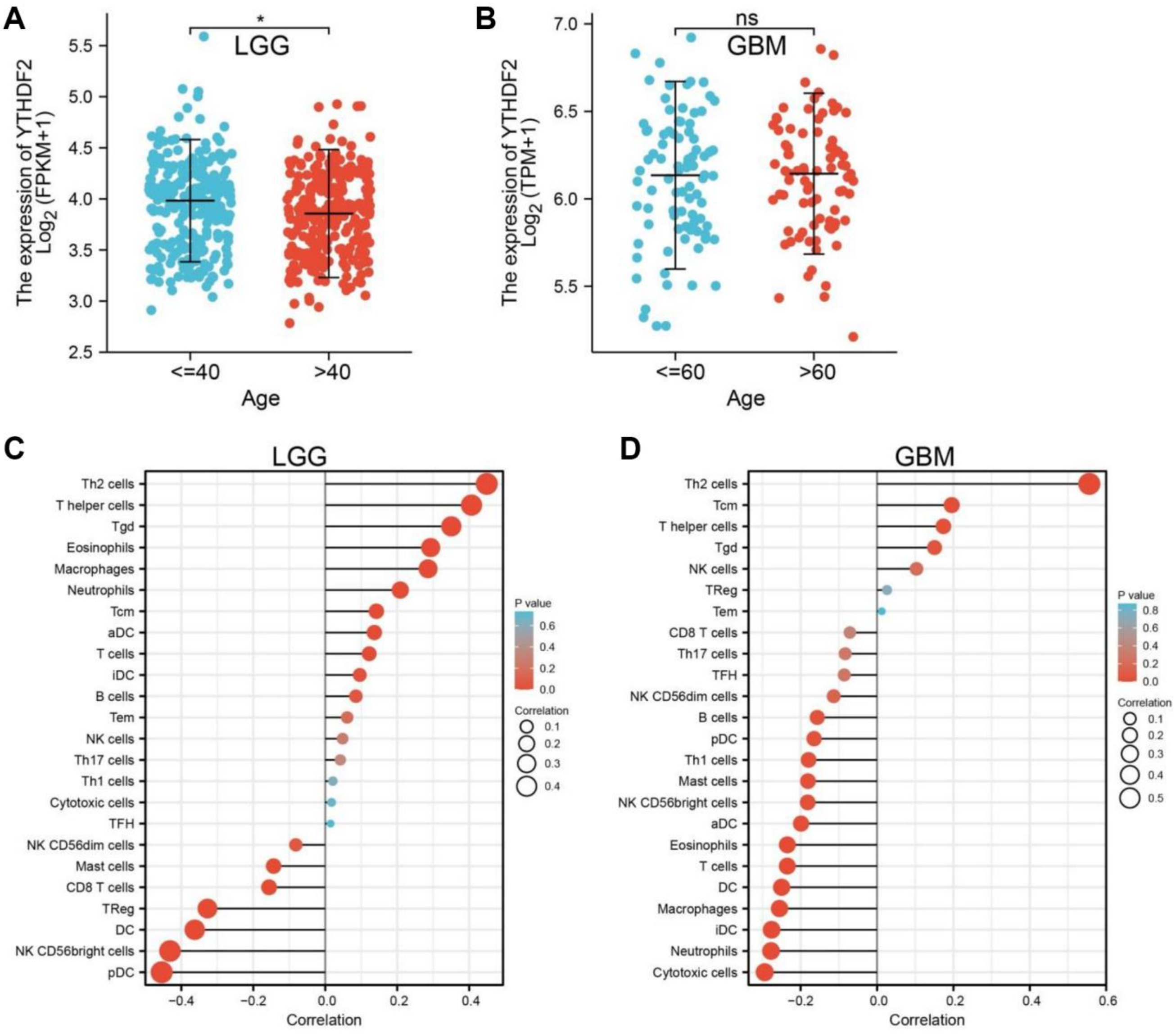

Supplementary Figure 1. Correlation between YTHDF2 expression and age in glioma patients. (**A**, **B**) Correlations between YTHDF2 expression and age in LGG and GBM patients (**C**, **D**) Correlation between YTHDF2 expression and immune infiltration in LGG and GBM datasets.
